# Editorial: Neurobehavioral Mechanisms of Reward: Theoretical and Technical Perspectives and Their Implications for Psychopathology

**DOI:** 10.3389/fnbeh.2022.967922

**Published:** 2022-07-05

**Authors:** George Panagis, Styliani Vlachou, Alejandro Higuera-Matas, Maria J. Simon

**Affiliations:** ^1^Laboratoy of Behavioral Neuroscience, Department of Psychology, University of Crete, Rethymno, Greece; ^2^Behavioural Neuroscience Laboratory, Neuropsychopharmacology Division, Faculty of Science and Health, School of Psychology, Dublin City University, Dublin, Ireland; ^3^Department of Psychobiology, School of Psychology, National University of Distance Education, Madrid, Spain; ^4^Department of Psychobiology, Mind, Brain and Behaviour Research Center (CIMCYC), University of Granada, Granada, Spain

**Keywords:** dopamine, reward, intracranial self-stimulation, motivation, choice, learning, animal models, psychopathology

For more than 50 years, research on brain mechanisms of reward has largely focused on the mesolimbic system and the release of dopamine in the nucleus accumbens when this system is activated. Numerous preclinical animal studies have electrically stimulated fibers of the medial forebrain bundle (MFB) at different localizations in combination with behavioral procedures (operant and/or classical conditioning), resulting in the (indirect) activation of this system (Gallistel et al., 1981; Milner, 1989; Wise and Rompré, 1989; Yeomans, 1990). This has also been achieved by drug self-administration or a mixture of drug and electrical self-stimulation (Fowler, 1999; Negus and Miller, 2014). These research lines have yielded a considerable body of knowledge with practical implications in the field of addictive behaviors, including drug abuse, food craving, and behavioral (or substance-free) addictions such as gambling, shopping, video gaming, and social networking, etc. (Antons et al., 2020). The dysfunction of brain reward systems has been reported in all of these cases as well as in individuals with neuropathological diseases such as depression or schizophrenia.

Since the 2000s, there has been a substantial increase in the study of the dissociation of specific components of these reward circuits. Authors have identified specific subsystems involved in motivation (seeking or wanting behaviors), hedonic-affective reactions (consummatory or liking behaviors), and learning processes (incentive learning, reward-related decision making, goal selection, etc.), some of which might be related to the activation of non-dopaminergic networks (Berridge and Robinson, 1998; Ikemoto and Panksepp, 1999; Salamone and Correa, 2002; Panksepp and Yovell, 2014; Morales and Margolis, 2017; Darcq and Kieffer, 2018). In fact, some researchers observed hedonic reactions in animals in the absence of dopamine (Grill and Norgren, 1978; Kirkpatrick and Fowler, 1989; Cannon and Palmiter, 2003), whereas individuals with high levels of synaptic dopamine were found to run faster toward goals and need fewer trials to learn an incentive runway task (Peciña et al., 2003). Hence, dopamine might be specifically related to motivation, seeking behavior and decision-making, an issue that still remains controversial (Adamantidis et al., 2011; Salamone et al., 2022).

In brain electrical self-stimulation (or intracranial self-stimulation), animals learn to press a lever connected to a cable, a stimulator, and a chronically implanted electrode that delivers electric pulses to activate specific brain regions. This behavioral procedure has proven especially useful to investigate circuits involved in motivational and decision-making components of the brain reward system. In this regard, some researchers have drawn on ideas from the psychophysical approach, investigating the link between the physical qualities of a stimulus and the inner psychological states of individuals in order to deepen our understanding of the relationship between seeking (operant) behaviors and the brain reward system (Fowler et al., 1986; Shizgal, 1997). The “Shift-Curve paradigm” has been used to explore how the amplitudes or frequencies of electrical stimulation can modify the response rate, generating a graphical representation of this sigmoidal function (Miliaressis et al., 1986; Carlezon and Chartoff, 2007). Other studies have investigated how drugs can produce a shift in the sigmoidal curve along the axis (Gallistel and Karras, 1984; Negus and Miller, 2014). This paradigm can lead to a “serial model” in which increases in amplitude or frequency drive instrumental behavior toward an asymptotic value (which means “*the more, the better*”) (Miliaressis et al., 1986; Carlezon and Chartoff, 2007).

More sensitive models have recently been developed, such as the “reward-mountain model”, which considers not only amplitude or frequency variations but also the “opportunity cost”. In the study by Pallikaras and Shizgal (this issue), the latter is related to the subjective effort made by an individual to press a lever for rewarding electrical stimulation when other “leisure” activities are simultaneously possible. In this way, the model includes a variable for the distribution of the time spent by animals between lever press and alternative behaviors (e.g., grooming, exploring, or resting). In addition, the effect of drugs (e.g., psychostimulants, DAT blockers, etc.) is considered an independent variable that can modulate thresholds and cause individuals to reevaluate the effort cost (Arvanitogiannis and Shizgal, 2008; Hernandez et al., 2012; Trujillo-Pisanty et al., 2013, 2020; Pallikaras et al., 2022). According to Pallikaras and Shizgal, this procedure represents an evolution from a “series” to a “convergent” model of causation, in which multiple factors may have a role in seeking and decision-making behaviors and can be represented in a tridimensional function called the “reward mountain”. This model may best fit the complex/paradoxical results obtained on the optogenetic activation of midbrain dopamine neurons. Accordingly, some of the bulk of fibers that form the MFB would be dopaminergic, but others would be neurochemically heterogeneous ascendant and descendent fibers that run in parallel and may have been underestimated by researchers to date.

The experimental study contributed by Velazquez-Martínez et al. (this issue) also utilizes electrical stimulation to study motivational mechanisms of the reward system. They explore functions relating the rate of lever-pressing for electrical stimulation of the lateral hypothalamus (which relays fibers from the MFB that sustain vigorous operant behaviors) to the intensity (amplitude) or frequency of stimulation in the shift-curve paradigm. They also consider the effort cost by introducing three fixed values of high, moderate, or low effort. The second part of their study uses rewarding electrical stimulation as a discriminative stimulus, with the animals having access to two levers for a reward program of high or low efficacy, respectively. Administration of the dopamine antagonist *pimozide* was found to interfere with the effort cost but to have no apparent effect when electrical stimulation was employed as a discriminative stimulus, in agreement with results obtained by other groups (Salamone et al., 2018). These findings support the original hypothesis that non-dopaminergic fibers passing through the MFB may be involved in processing some other components of reward.

Translational studies have attempted to determine, by the intensive examination of laboratory models, the relevance of functional and/or neurochemical changes in the neurobiological subsystems involved in reward to the clinical treatment of motivational and/or hedonic alterations in patients with neuropathological disorders such as depression or schizophrenia.

Various therapeutic approaches have been adopted toward patients with depression, including psychomotor stimulant-based monotherapy, “behavioral activation” (a psychotherapeutic tool that aims to engage patients in reward-seeking activities and the avoidance of punishments), and deep-brain stimulation of the MFB (Tindall et al., 2017; Coenen et al., 2021). Failures with the monotherapy alongside successes achieved with stimulation in cases of drug-resistant depression suggest the involvement of both non-dopaminergic and dopaminergic fibers, as proposed by the “convergence model”, which could be activated by deep stimulation. However, novel techniques are required to reveal their neurochemical substrates, allowing the development of more targeted therapeutic strategies. At any rate, according to Pallikaras et al. (2022), animal self-stimulation models remain a valuable tool to improve our understanding of the underlying neural and psychological mechanisms.

For their part, Abram et al. (this issue) point out that different psychological factors can modulate the functioning of reward circuits in individuals with schizophrenia, whose balance between positive and negative emotions is less favorable to the former, lowering their hedonic perception (Strauss et al., 2017, 2020). However, they also appear to show deficits in reward anticipation, related to both prospection (capacity to imagine future experiences) and episodic memory (recall of past experiences), while motivation may also be affected. Certain personality features (optimism vs. pessimism) alongside a propensity for negative thoughts about themselves, others, and the future (part of the plethora of negative symptoms of schizophrenia), rumination (a positive symptom), and factors related to the social setting (e.g., sensitivity to social reward) may play an important role in the dysfunction of reward systems observed in people with a diagnosis of schizophrenia (amotivation, anhedonia, etc.). Further research is needed to elucidate these aspects and to explore the effects on these neurobiological systems of different clinical interventions, including “cognitive reappraisal”, “mindfulness”, and “savoring”. The results could facilitate the development of novel therapeutic tools.

Alterations in reward-related decision making are also observed in relation to the substance use disorders and behavioral addictions as shown in the study by Schluter and Hodgins on gambling disorders (this issue), which include both impulsive behaviors and the choice of risk. They describe a maintained impulsivity in individuals whether or not they have a gambling disorder, whereas the propensity for risk is only observed while this is responsible for sustaining gambling behavior.

In summary, various basic and translational investigations, including those gathered in this issue and many others, are beginning to find common ground on the need to dissociate specific components of the brain reward system and their relationship with behavior. Although they mostly derive from very different perspectives (pharmacological, behavioral, genetic, epigenetic, computational…), the results of these studies are beginning to fit together like the pieces of a puzzle, revealing a complex picture of the functioning of brain reward mechanisms. It shows that the mesolimbic dopaminergic system continues to play a key part (as a “*primum inter pares”*) but other systems also have a role. This supports the design of better-targeted treatments of the different neuropathological disorders in which these systems are altered.

## Author Contributions

All authors listed have made a substantial, direct, and intellectual contribution to the work and approved it for publication.

## Conflict of Interest

The authors declare that the research was conducted in the absence of any commercial or financial relationships that could be construed as a potential conflict of interest.

## Publisher's Note

All claims expressed in this article are solely those of the authors and do not necessarily represent those of their affiliated organizations, or those of the publisher, the editors and the reviewers. Any product that may be evaluated in this article, or claim that may be made by its manufacturer, is not guaranteed or endorsed by the publisher.
